# Neprilysin and Aβ Clearance: Impact of the APP Intracellular Domain in NEP Regulation and Implications in Alzheimer’s Disease

**DOI:** 10.3389/fnagi.2013.00098

**Published:** 2013-12-23

**Authors:** Marcus O. W. Grimm, Janine Mett, Christoph P. Stahlmann, Viola J. Haupenthal, Valerie C. Zimmer, Tobias Hartmann

**Affiliations:** ^1^Experimental Neurology, Saarland University, Homburg, Saar, Germany; ^2^Neurodegeneration and Neurobiology, Saarland University, Homburg, Saar, Germany; ^3^Deutsches Institut für DemenzPrävention, Saarland University, Homburg, Saar, Germany

**Keywords:** Alzheimer’s disease, Abeta degradation, neprilysin, AICD, amyloid beta, transcriptional regulation, Abeta clearance

## Abstract

One of the characteristic hallmarks of Alzheimer’s disease (AD) is an accumulation of amyloid β (Aβ) leading to plaque formation and toxic oligomeric Aβ complexes. Besides the *de novo* synthesis of Aβ caused by amyloidogenic processing of the amyloid precursor protein (APP), Aβ levels are also highly dependent on Aβ degradation. Several enzymes are described to cleave Aβ. In this review we focus on one of the most prominent Aβ degrading enzymes, the zinc-metalloprotease Neprilysin (NEP). In the first part of the review we discuss beside the general role of NEP in Aβ degradation the alterations of the enzyme observed during normal aging and the progression of AD. *In vivo* and cell culture experiments reveal that a decreased NEP level results in an increased Aβ level and vice versa. In a pathological situation like AD, it has been reported that NEP levels and activity are decreased and it has been suggested that certain polymorphisms in the NEP gene result in an increased risk for AD. Conversely, increasing NEP activity in AD mouse models revealed an improvement in some behavioral tests. Therefore it has been suggested that increasing NEP might be an interesting potential target to treat or to be protective for AD making it indispensable to understand the regulation of NEP. Interestingly, it is discussed that the APP intracellular domain (AICD), one of the cleavage products of APP processing, which has high similarities to Notch receptor processing, might be involved in the transcriptional regulation of NEP. However, the mechanisms of NEP regulation by AICD, which might be helpful to develop new therapeutic strategies, are up to now controversially discussed and summarized in the second part of this review. In addition, we review the impact of AICD not only in the transcriptional regulation of NEP but also of further genes.

## Alzheimer’s Disease

Alzheimer’s disease (AD) is one of the most common neurodegenerative disorders of the central nervous system. Currently more than 35 million people are affected worldwide and the number of affected people is estimated to double every 20 years leading to more than 115 million AD cases in the year 2050 (AD International, 2013). It is characterized by a degeneration of neurons in multiple brain regions, mainly the cortical and subcortical areas and hippocampus, leading to a loss of cognitive brain functions, memory impairment, and often to behavioral and physiological changes like apathy and depression. Characteristic histopathological hallmarks are intracellular neurofibrillary tangles composed of a hyperphosphorylated form of the microtubule-associated protein tau and extracellular β-amyloid plaques in brain tissue (Grundke-Iqbal et al., 1986; Selkoe, 2004; Binder et al., 2005). Major components of the β-amyloid deposits are hydrophobic amyloid-β-peptides (Aβ), which are 38–43 amino acids (aa) long fragments derived from proteolytic processing of the amyloid precursor protein (APP) (Glenner and Wong, 1984; Masters et al., 1985; Aguzzi and Haass, 2003).

Most of the AD cases belong to the sporadic form of the disease with an onset after the age of 65 (late onset AD, LOAD). Less than 5% of all AD cases are genetically based (familial AD, FAD) due to mutations in the genes encoding for APP or presenilin1 (PS1) and presenilin2 (PS2), proteins involved in the proteolytic cleavage of APP, leading to an earlier age of onset (Scheuner et al., 1996; Hardy, 1997). According to the amyloid cascade hypothesis, the excessive accumulation and aggregation of the 4 kDa Aβ-peptide is regarded to be central in the pathogenesis of AD, initiating cellular cascades leading to synaptic loss and neuronal injury (Hardy and Higgins, 1992; Hardy and Selkoe, 2002). Especially an increase in Aβ42 (indicating 42 aa) is reported to trigger the disease process due to its high tendency to aggregate. Most of the FAD-linked mutations lead to an increase in total Aβ levels or the ratio of Aβ42/Aβ40 resulting in an aggressive and early occurring pathology (Kowalska, 2004; Duering et al., 2005). Accumulating evidence suggests that oligomeric Aβ species including dimers and trimers (Cleary et al., 2005; Shankar et al., 2007), small diffusible oligomers (Lambert et al., 1998), donut-like annular oligomers (Lashuel et al., 2002), and large amylospheroids (Hoshi et al., 2003) represent the most toxic forms of the peptide causing impaired synaptic and neuronal functions (Haass and Selkoe, 2007; Walsh and Selkoe, 2007).

The Aβ levels in brain, are not only dependent on the *de novo* synthesis by APP processing, but also by its elimination via different mechanisms including its proteolytic degradation, transport processes, cell mediated clearance, and its deposition into insoluble aggregates. While enhanced Aβ generation and a shift in the Aβ40/42 ratio have been shown to be associated with FAD, a diminished Aβ clearance has been long hypothesized to predominate in LOAD (Tanzi et al., 2004; Hama and Saido, 2005). Indeed, recently a study by Mawuenyega et al. (2010) confirmed a significant impairment in the clearance of cerebrospinal fluid (CSF) Aβ in LOAD patients.

## APP and the Generation of Aβ

### The APP family of proteins

Amyloid precursor protein is a ubiquitously expressed type I integral transmembrane protein consisting of a large ectodomain, one single transmembrane domain, and a short intracellular tail (Kang et al., 1987; Dyrks et al., 1988). It belongs to a small gene family including the APP-like proteins 1 and 2 (APLP1 and APLP2) in mammals (Sprecher et al., 1993; Wasco et al., 1993), APL-1 in *C. elegans*, APPL in *D. melanogaster*, and APPa and b in zebrafish (Musa et al., 2001). While APP and APLP2 are ubiquitously expressed, APLP1 expression is restricted to neurons (Tanzi et al., 1988; Slunt et al., 1994; Lorent et al., 1995). The gene encoding for human APP is located on chromosome 21 and contains 18 exons (Yoshikai et al., 1990). Alternative splicing of exons 7 and 8 generates APP mRNAs encoding for several isoforms, mainly APP770, APP751, and APP695 (referring to length in aa) with the latter being the major neuronal species (Sandbrink et al., 1994). While single knockout (KO) of one of the APP family members results only in viable mild phenotypes, APP/APLP2 double KO animals are perinatal lethal showing for example severe deficits in neuromuscular junctions (Wang et al., 2005a). Mice genetically deleted of all three APP gene family members show a neuronal ectopy in forebrain resembling the human type 2-lissencephaly, which also leads to animal death short time after birth (Herms et al., 2004). These severe phenotypes indicate important, partially overlapping physiological functions of APP, APLP1, and APLP2 in the mammalian nervous system (Aydin et al., 2012).

### Proteolytic processing of APP

**Figure 1:**
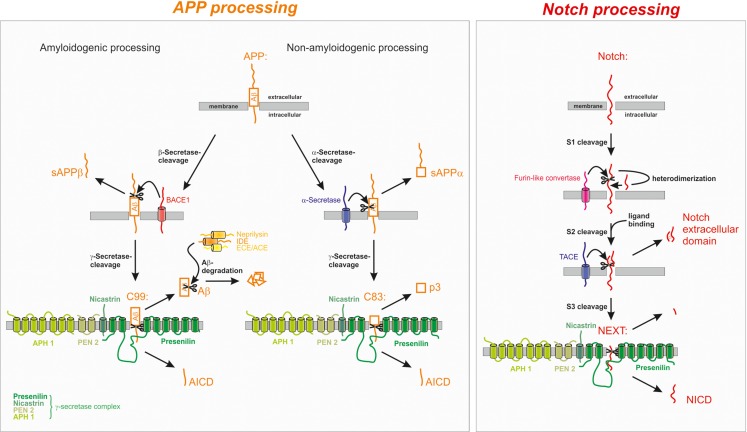
**Proteolytic processing of the amyloid precursor protein (APP) and Notch**. APP processing: in the amyloidogenic processing pathway, APP is cleaved by the β-secretase BACE1 generating C99 which is further cleaved to the Amyloid-β peptide (Aβ). The non-amyloidogenic processing pathway is initiated by α-secretase cleavage of APP within the Aβ domain, thus precluding the generation of Aβ. α- and β-secretase cleavage releases the soluble forms of APP, sAPPα, and sAPPβ, respectively, into the extracellular space. The remaining membrane-bound C-terminal fragments C83 and C99 are further processed by the γ-secretase complex leading to the generation of the non-toxic p3 from C83 or of the amyloidogenic Aβ peptide from C99. Aβ is rapidly degraded by several enzymes, for example neprilysin (NEP), insulin-degrading enzyme (IDE), endothelin-converting enzyme (ECE), and angiotensin converting enzyme (ACE). In both processing pathways the APP intracellular domain (AICD) is released into the cytosol. Notch processing: after maturation S1 cleavage of the Notch receptor precursor by a furin-like convertase, Notch is processed similarly to APP. Ligand binding triggers the S2 cleavage by the α-secretase TACE/ADAM17 leading to the release of the Notch extracellular domain into the extracellular space. The remaining membrane-bound fragment Notch extracellular truncation (NEXT) is further processed by the γ-secretase complex resulting in the release of the Notch intracellular domain (NICD) from the membrane.

Proteolytic processing of APP occurs via two different cleavage pathways and shows large homologies to the proteolytic processing of the Notch receptor as demonstrated in Figure 1.

In the case of the amyloidogenic processing pathway, APP is first cleaved within the extracellular domain by the transmembrane aspartyl protease BACE1 (β-site APP cleaving enzyme 1) shedding off the soluble ectodomain sAPPβ and generating the membrane-tethered C-terminal fragment (CTF) C99 (termed according to length in aa) (Vassar et al., 1999). In the case of the predominant non-amyloidogenic APP cleavage cascade the soluble ectodomain sAPPα and the C-terminal membrane-spanning stub C83 are generated by the activity of the α-secretases, members of the ADAM (a disintegrin and metalloprotease) protein family (Lammich et al., 1999; Hooper and Turner, 2002). The α-secretase cleavage site is located at position 16 within the Aβ sequence, precluding the generation of Aβ in the non-amyloidogenic pathway. The two alternative pathways are located in different subcellular compartments: while non-amyloidogenic APP processing, which is the major pathway of APP cleavage in all cells, takes place at the plasma membrane (Parvathy et al., 1999; Ehehalt et al., 2003), the amyloidogenic APP processing by β-secretase cleavage takes place in endosomes having a more acidic pH, which is also the pH optimum of BACE1 mediated APP turn over (Grbovic et al., 2003; Carey et al., 2005; Rajendran et al., 2006).

The APP-CTFs are subsequently cleaved by γ-secretase liberating either the non-toxic peptide p3 (from C83) or Aβ (from C99) and the APP intracellular domain (AICD), which is discussed to regulate the expression of several genes, into the cytosol (Haass et al., 1992; Passer et al., 2000; Cao and Sudhof, 2004). The γ-secretase complex consists of at least four proteins, the proteins PS1 or PS2 as catalytic core, nicastrin, Aph (anterior pharynx defective) 1a or b, and presenilin enhancer 2 (PEN2) (Grimm et al., 2002; Baulac et al., 2003; Edbauer et al., 2003; Kimberly et al., 2003). It cleaves its substrates within the hydrophobic environment of the membrane, a process frequently involved in important signaling pathways and termed regulated intramembrane proteolysis (RIP) (Lichtenthaler et al., 2011). As γ-secretase cleavage can take place at different positions, Aβ and AICD peptides vary in length. Since the major produced Aβ species end at position 40 or 42 (Haass et al., 1992; Seubert et al., 1992; Roher et al., 1993; Iwatsubo et al., 1994), one can assume that AICD should begin at position 41 or 43 (aa numbers referring to the Aβ sequence). Contrary, most of the generated AICD seems to begin close to aa position 50 (Gu et al., 2001; Yu et al., 2001). This might be explained by the finding of another cleavage site of APP, termed ϵ-cleavage. It takes place not in the middle of the transmembrane domain of APP, but close to the cytoplasmic face of the plasmamembrane. Interestingly, ϵ-cleavage shares similarities to several other cellular proteolytic processes as for example the Notch-site-3 cleavage (Sastre et al., 2001; Weidemann et al., 2002). Presumably, processing of the CTF of APP at this cleavage site is carried out by the γ-secretase complex. It was shown that γ-secretase-inhibitors preventing Aβ40 and Aβ42 production also abolished AICD generation resulting from ϵ-cleavage. In addition to that, production of ϵ-cleaved AICD depends directly on the expression of PS1 (Yu et al., 2001). Up to now, the exact underlying mechanism how γ-secretase manages to cleave one single protein at different sites still has to be elucidated. It is discussed that cleavage occurs at aa 40 or 42 first, followed by aminopeptidase action on the 57 or 59 aa long AICD peptides, leading to the generation of truncated AICD (50-99 aa) (Gu et al., 2001; Chavez-Gutierrez et al., 2012). Another explanation is given by a model, in which APP is first cleaved at aa 49 or 50 resulting in the generation of AICD (50-99 aa) and the corresponding Aβ counterparts Aβ1–48 and Aβ 1–49 (Qi-Takahara et al., 2005) which could possibly be further truncated by carboxypeptidase activity (Funamoto et al., 2004). The stepwise degradation of Aβ then results in different Aβ species (38–43 aa in length) (Chavez-Gutierrez et al., 2012). Alternatively, it is discussed that γ-secretase possibly cleaves APP simultaneously at several sites or that other proteases are involved in this process. Yu et al. (2001) proposed the model of a membrane-resident, multicatalytic protease introducing non-selectively the cleavage of membrane proteins by resident enzymes. Caspase-3 is also reported to truncate AICD and hence to produce the 31 aa peptide C31 (Gervais et al., 1999), which was shown to be elevated in AD brains and to be involved in cell death pathways (Lu et al., 2000).

The homologs APLP1 and APLP2, lacking the Aβ region, are processed similarly to APP by α-, β-, and γ-secretase cleavage generating the APLP intracellular domains ALID1 and ALID2 (Walsh et al., 2003; Eggert et al., 2004).

## The Aβ Degrading Zinc-Metalloprotease Neprilysin

### Mechanisms of Aβ clearance in brain

As already mentioned, the Aβ levels in brain depend not only on Aβ production, but also on its removal via different clearance pathways and enzymatic degradation. Under physiological conditions the peptide is rapidly cleared from brain by a combination of several mechanisms, resulting in a half-life of approximately 1–2, 5 h (Savage et al., 1998; Cirrito et al., 2003). Aβ can be exported from brain across the blood-brain-barrier by the lipoprotein receptor-related protein (LRP) and by the P-glycoprotein efflux pump (pgP/MDR1/ABCB1) (Kang et al., 2000; Shibata et al., 2000; Lam et al., 2001), whereas the reentry of circulating Aβ from blood to brain is mainly mediated by RAGE (receptor for advanced glycation end products) (Deane et al., 2003). In plasma the peptide is bound by a soluble form of LRP (sLRP) and transported to liver and kidneys, which mediate the systemic clearance of unbound Aβ and of sLRP-Aβ complexes (Sagare et al., 2007, 2012). Furthermore, phagocytosis by microglia followed by lysosomal degradation and perivascular drainage along basement membranes into the CSF contributes to Aβ removal from brain (Rogers and Lue, 2001; Preston et al., 2003). A diversity of enzymes is capable of cleaving Aβ at a single or at multiple sites, most of them are metalloproteases differing in their regional and subcellular distribution, their pH-optima and their ability to degrade the different species of the peptide (Miners et al., 2011; Saido and Leissring, 2012). Some Aβ degrading enzymes are localized within the secretory pathway and catabolize intracellular Aβ prior to its secretion into the extracellular space (Eckman et al., 2001; White et al., 2006). Aβ40 is discussed to be mainly degraded intracellularly, whereas Aβ42 is basically degraded outside the cell (Hama et al., 2004). Therefore one might speculate that the different intra- and extracellular pools of Aβ are not removed by one single protease, but rather by several enzymes working cooperatively together (Saido and Leissring, 2012). Aβ exists in a dynamic equilibrium of soluble monomeric, oligomeric, and fibrillar forms (Dahlgren et al., 2002). Fibrillization is dependent on the formation of β-sheet-structures between the residues 18 and 42 making this region less accessible to proteolytic cleavage (Crouch et al., 2009). Therefore, all known Aβ degrading enzymes are capable of cleaving monomeric Aβ, but most of them show a restricted ability to degrade oligomeric or fibrillar species of the peptide. The proteolysis of Aβ is generally assumed to be beneficial, but for most of the resulting products their neurotoxic potential or potential physiological relevance still has to be investigated.

#### Summary

Aβ levels in brain are influenced not only by Aβ production, but also by different clearance mechanisms including its clearance to blood and CSF, phagocytosis by microglia, and enzymatic degradation. The zinc-metalloprotease neprilysin (NEP) is one of the most prominent Aβ degrading enzymes.

### General features of NEP

It has been shown that a neutral endopeptidase sensitive for thiorphan and phosphoramidon plays a key role in Aβ42 catabolism in rat brain, leading to the proposal that NEP is the major Aβ degrading peptidase *in vivo* (Iwata et al., 2000). In line with these results NEP deficiency results in twofold elevated levels of endogenous Aβ40 and Aβ42 in different brain regions and in defects in the degradation of exogenously administered Aβ42 (Iwata et al., 2001). Today NEP is one of the major and best characterized Aβ degrading enzymes (Hersh and Rodgers, 2008).

Neprilysin is also named CALLA (common acute lymphocytic leukemia (ALL) antigen), enkephalinase, neutral endopeptidase 24.11, and CD10 antigen (Brown et al., 1974; Schwartz et al., 1980; Letarte et al., 1988). It belongs to the family of M13 zinc-metalloendopeptidases and is an ubiquitously occurring type II integral membrane protein consisting of 742 aa with a molecular weight ranging from 85 to 110 kDa depending on differences in its glycosylation (Relton et al., 1983; Malfroy et al., 1988). It is highly expressed in kidney, but also in other tissues like brain (Erdos and Skidgel, 1988). The active center of the enzyme faces the extracellular side of the membrane, an ideal topology for the degradation of peptides located in the extracellular space or associated to the membrane (Fukami et al., 2002).

The human NEP gene [MME, epicatechin (EC) 3.4.24.11] maps to chromosomal region 3q25.1–q25.2 and is composed of 24 exons, highly conserved among mammals (D’Adamio et al., 1989). The expression of the NEP gene is controlled by at least two different promoters and varies between different tissues. Alternative splicing in the 5′-untranslated region results in four separate mRNA transcripts without affecting the coding region (Li et al., 1995). The protein consists of a short N-terminal cytosolic region, a single transmembrane helix and a large extracellular domain containing the typical HEXXH zinc binding motif that is essential for the proteolysis of its various substrates described below (Barnes et al., 1995; Turner et al., 2001), preferentially oligopeptides consisting of up to 40 aa (Oefner et al., 2000). NEP is involved in neuropeptide signaling and in the regulation of vascular tone (Roques et al., 1993). Moreover, it is used as an important cell-surface marker in the diagnosis of human acute lymphocytic leukemia (ALL) (Brown et al., 1975) and reported to play a role in the progression of several other cancers (Gohring et al., 1998; Papandreou et al., 1998). In the central nervous system NEP is mainly expressed by neurons (Matsas et al., 1986), but also by activated astrocytes and microglia (Fisk et al., 2007; Hickman et al., 2008). In neurons NEP is subcellularly localized along axons and synapses (Fukami et al., 2002) where NEP mediated Aβ degradation mainly takes place (Barnes et al., 1992; Hama et al., 2001; Iwata et al., 2004). This subcellular localization underlines the role of NEP in the degradation of several neuropeptides, for example enkephalins, substance P, neuropeptide Y, tachykinins, bradykinin, and somatostatin (Matsas et al., 1984; Roques et al., 1993; Barnes et al., 1995). The *in vivo* functions of NEP have been analyzed by utilizing NEP gene disrupted mice. These animals show enhanced lethality to endotoxin treatment, probably due to the role of NEP in the metabolism of pro-inflammatory peptides, lower blood pressure, and higher microvascular permeability (Lu et al., 1995).

#### Summary

Neprilysin is an 85–110 kDa zinc-dependent membrane metalloprotease degrading numerous extracellular located substrates, among them Aβ and several neuropeptides. The enzyme is expressed in various tissues including the central nervous system, where it is mainly present in neurons with a subcellular localization along axons and synapses.

### NEP and its function in Aβ degradation

**Figure 2:**
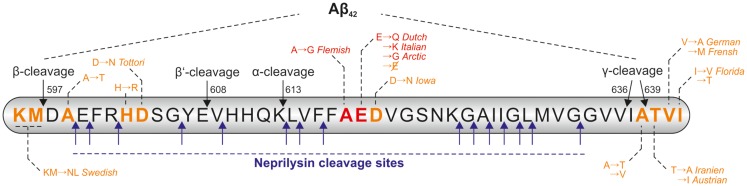
**Cleavage sites of NEP within the Aβ-sequence and positions of FAD mutations**. Within the Aβ sequence there are several cleavage sites for neprilysin (NEP), the cleavage positions of α-, β-, and γ-secretase are also indicated. Some of the pathological point mutations within the Aβ sequence are reported to result in a higher resistance of the peptide to NEP-catalyzed proteolysis (highlighted in red), other known mutation sites are shown in orange. The amino acid numbers are referring to the APP695 isoform.

The ability of NEP to cleave Aβ monomers *in vitro* as well as in cell culture was analyzed in different studies (Howell et al., 1995; Hama et al., 2001; Kanemitsu et al., 2003; Marr et al., 2003). It is worth mentioning that NEP is reported to cleave monomeric Aβ40 more efficiently than Aβ42. In an *in vitro* degradation assay only 27% of the added monomeric Aβ42 was degraded by NEP, in contrast the enzyme cleaved 73% of the added Aβ40 monomers (Kanemitsu et al., 2003). In the meantime several cleavage sites of NEP within the Aβ sequence have been identified (Carson and Turner, 2002; Wang et al., 2006; Miners et al., 2011), which are summarized in Figure 2. The ability of the enzyme to degrade Aβ oligomers and fibrils is controversially discussed, e.g., NEP seems to be capable of degrading synthetic oligomers formed non-enzymatically from synthetic Aβ40 and Aβ42 (Kanemitsu et al., 2003), but not oligomers naturally secreted from cells (Leissring et al., 2003).

Interestingly, some of the pathogenic APP mutations result in a higher resistance of Aβ to NEP-catalyzed proteolysis. Tsubuki et al. incubated wildtype Aβ40 and Aβ40 peptides containing the Dutch, Flemish, Italian, and Arctic mutation (Van Broeckhoven et al., 1990; Hendriks et al., 1992; Nilsberth et al., 2001; Bugiani et al., 2010) (indicated in Figure 2) with purified mouse NEP prior to analysis of the peptides by high-performance liquid chromatography (HPLC). All mutated Aβ peptides were more resistant to NEP-catalyzed cleavage in comparison to wildtype Aβ40 (Tsubuki et al., 2003). Betts et al. analyzed the same peptides and found significant differences in their tendency to aggregate. Furthermore, they demonstrated aggregated wildtype Aβ40 to be less well degraded than its monomeric form. Proteolysis analysis of the mutated monomeric peptides by HPLC revealed that all mutated Aβ species with exception of the Flemish mutated peptide are degraded equally well by NEP as wildtype Aβ. Only Aβ bearing the Flemish mutation was degraded significantly more slowly by NEP. The authors concluded, that the most likely explanation for this divergence in results lies in differences in the aggregation state of the analyzed peptides (Betts et al., 2008).

The *in vivo* function of NEP in Aβ degradation reported by Iwata et al. has been verified in several other studies. It was demonstrated that intraneuronal Aβ42 deposits and Aβ42 induced neuronal loss can be sufficiently suppressed in transgenic *D. melanogaster* expressing human NEP and Aβ42 (Iijima-Ando et al., 2008). Moreover, KO of NEP in mice result in an increase in the levels of soluble and oligomeric Aβ leading to impaired synaptic plasticity and cognitive abnormalities in APP transgenic and wildtype animals (Huang et al., 2006; Madani et al., 2006). Conversely, overexpression of NEP in AD mouse models by using either genetic or viral approaches leads to decreased cerebral Aβ levels, inhibition of plaque formation, and enhanced life expectancy (Leissring et al., 2003; Marr et al., 2003; Iwata et al., 2004; Poirier et al., 2006). In line with this, improved behavioral performance and cognitive functions in the NEP overexpressing animals is reported (Poirier et al., 2006; El-Amouri et al., 2008; Spencer et al., 2008). Recently, Iwata et al. (2013) demonstrated the AAV vector-mediated NEP gene transfer into an AD mouse model to significantly reduce monomeric, dimeric, trimeric, and tetrameric forms of Aβ accompanied by alleviation of abnormal learning and memory function. Further, the administration of recombinant soluble NEP by intracerebral injection into AD mice was reported to result in significantly reduced accumulation of Aβ and additionally in improved behavioral performance on the water maze test (Park et al., 2013). Conversely, Meilandt et al. (2009) failed to assess behavioral improvement in the Morris water maze test and reduction of Aβ oligomers in hAPP/NEP double transgenic animals despite 50% reduction in soluble Aβ levels and prevention of plaque formation, demonstrating the inability of NEP to cleave some naturally occurring oligomeric Aβ species.

Furthermore, NEP is discussed to be a genetic risk factor for the development of AD. It has been demonstrated that individuals with certain polymorphisms in the NEP gene have an increased risk for AD (Helisalmi et al., 2004) and the susceptibility to AD is even higher when insulin-degrading enzyme (IDE), another Aβ-degrading enzyme, also shows a polymorphism (Vepsalainen et al., 2009). In contrast, other studies reported a lack of association between NEP polymorphisms and the risk for developing AD (Sodeyama et al., 2001; Oda et al., 2002; Wood et al., 2007).

#### Summary

While NEP is capable to cleave Aβ monomers *in vitro* and *in vivo*, its ability to degrade oligomeric Aβ species is controversially discussed. Decreased NEP levels were shown to result in increased Aβ levels, impaired synaptic plasticity, and cognitive abnormalities in WT and APP transgenic mice. Conversely, enhanced NEP levels in AD mouse models lead to reduced Aβ levels accompanied by an improvement in some behavioral tests.

## Altered NEP Regulation in Aging and AD

As already mentioned, defective Aβ clearance has been long hypothesized to predominate in LOAD. Therefore, a possible correlation of NEP levels with the progression of AD and with normal aging has been intensively investigated.

### Reduction of NEP levels in AD

In general, NEP seems to be reduced in brain areas early affected in AD and characterized by extensive plaque load. NEP levels are decreased in hippocampus (Yasojima et al., 2001a; Carpentier et al., 2002; Miners et al., 2006), temporal gyrus (Yasojima et al., 2001b), and cortex (Akiyama et al., 2001; Russo et al., 2005; Wang et al., 2005b; Miners et al., 2006) in human *post mortem* AD brains. NEP is also reduced in brain vasculature of AD patients (Carpentier et al., 2002; Miners et al., 2006) implicating a role of NEP in cerebral amyloid angiopathy (CAA). Moreover, alterations in NEP expression in AD are not restricted to brain tissue since the enzyme level was found to be also affected in CSF. In patients with mild cognitive impairment (MCI), substantial reduction of CSF NEP activity was observed followed by an elevation along with the progression of AD, suggesting that presynaptically located NEP is released into CSF as a consequence of synaptic disruption (Maruyama et al., 2005). An inverse relationship between NEP levels and Aβ accumulation has been shown in temporal and frontal cortex (Hellstrom-Lindahl et al., 2008) and in the vasculature (Carpentier et al., 2002) of human AD patients as well as in the hippocampus of APP transgenic mice (Fukami et al., 2002), indicating that even a slight reduction in NEP levels for several decades can lead to increased Aβ levels. However, other studies reported NEP levels in cortex not to be significantly altered (Hellstrom-Lindahl et al., 2008), or even elevated (Miners et al., 2009) in human *post mortem* AD brains compared to control brains. In the latter study NEP protein levels and activity, which was measured by the use of a highly specific immunocapture-based fluorometric activity assay (Miners et al., 2008), were reported to positively correlate with Braak stage. Interestingly, in this study NEP level was normalized to a protein marker for neuronal integrity, the neuron specific enolase (NSE). Moreover, quite recently Zhou et al. (2013) reported a decrease of NEP levels in cytoplasm and in contrast an increase in membrane fractions of MCI and AD brains indicating an altered subcellular localization of NEP in AD, which might also explain differences found in literature. Keeping in mind that NEP is a plasma membrane ectoenzyme, the detection of apparently cytoplasmatic NEP in this study is questionable and might possibly be an artifact resulting from the extraction process. In astrocytes the expression of NEP is upregulated in AD, especially in plaque-surrounding reactive astrocytes as demonstrated for aged APPswe mice (Apelt et al., 2003) and for human AD brains (Carpentier et al., 2002), further suggesting a possible role of astrogliosis in Aβ degradation.

Possible explanations for this divergence in results are for example methodological differences, differences in the analyzed brain areas, cell types and in sample preparation. Moreover, there are severe difficulties in specifically measuring NEP activity which could lead to different results. Taking in consideration that possibly a late upregulation of NEP in association with disease progression exists, difference in disease state of the analyzed individuals is a critical point for comparing the results of several studies. Additionally, indication exists that normal aging processes can also result in a reduction of NEP further underlying the importance of comparing stringently age matched controls to *post mortem* AD brains.

#### Summary

Neprilysin seems to be reduced in brain areas early affected in AD and characterized by high plaque load. A decline of NEP levels in AD has been observed in most of the studies although some authors assessed converse results.

### Reduction of NEP levels during aging

Several studies reported significant reductions of brain NEP levels in aged animals and human beings. NEP mRNA levels decline during aging in *D. melanogaster* brains (Iijima-Ando et al., 2008), similar effects were observed in mammals. NEP levels or enzyme activity diminish as a function of age in mouse cerebral cortex (Apelt et al., 2003), hippocampus (Caccamo et al., 2005), in whole mouse brain homogenate (Carter et al., 2006), and in the hippocampus of aged rats (Briones and Darwish, 2012). Iwata et al. (2002) further demonstrated by immunohistochemical analysis of the hippocampus of APP transgenic mice that NEP levels are selectively decreased in nerve terminals and axons upon aging. In line with these observations, a negative correlation between brain NEP levels and age was reported for both non-demented persons and AD patients (Russo et al., 2005; Hellstrom-Lindahl et al., 2008; Miners et al., 2009).

#### Summary

Neprilysin levels seem to be reduced during aging as demonstrated in aged *D. melanogaster*, mice, rats, and human beings.

### Mechanisms of NEP regulation

Taking into consideration that NEP expression and activity seem to be reduced in AD, it can be speculated that increased upregulation of NEP might have beneficial effects. The pathways involved in the regulation of NEP expression and activity possess an attractive therapeutic potential and have been further elucidated. Saito et al. reported that the neuropeptide somatostatin, which is a NEP substrate (Barnes et al., 1995), is able to upregulate NEP activity indicating a regulatory feedback cycle. Treatment of primary neurons with somatostatin results in a higher, somatostatin deficiency in mice in a reduced NEP activity, respectively (Saito et al., 2005). Pharmacological activation of the somatostatin receptor subtype-4 increases NEP activity in cortical tissue, suggesting that the somatostatin receptors are interesting pharmacological targets for the regulation of enzyme activity (Sandoval et al., 2012). In addition, NEP expression can be upregulated by the hormone estrogen in an estrogen receptor α and β dependent manner in human SH-SY5Y cells (Liang et al., 2010). A decrease of NEP activity combined with elevated brain Aβ levels were observed in ovariectomized animals with estrogen treatment reversing the effects (Petanceska et al., 2000; Huang et al., 2004). We and others have shown the secosteroid vitamin D to be involved in the regulation of NEP *in vitro* and *in vivo*. Vitamin D deficiency in mice results in a lowered NEP expression and enzyme activity, while vitamin D supplementation elevates NEP levels in cultured cells and in the brain of aged rats (Briones and Darwish, 2012; Grimm et al., 2013). The reported downregulation of somatostatin (Davies et al., 1980; Lu et al., 2004; Gahete et al., 2010), estrogen (Manly et al., 2000; Barron and Pike, 2012), and vitamin D (Annweiler et al., 2011; Llewellyn et al., 2011) in aged individuals and AD patients may explain the downregulation of NEP upon aging and in AD. Furthermore, oxidative stress, which is increased in AD brain, leads to decreased half-life of NEP and decreased enzyme activity (Wang et al., 2003; Shinall et al., 2005). Oxidative stress initiates the formation of 4-hydroxy-non-enal (HNE), a by-product of lipid peroxidation reported to be increased in AD brains, interacting with, and inactivating a variety of enzymes including NEP (Lauderback et al., 2001; Wang et al., 2003). In cell culture studies using SK-N-SH cells, green tea extract (Melzig and Janka, 2003), and more specifically the antioxidative green tea polyphenols EC, epigallocatechin (EGC), and epigallocatechin gallate (EGCG) among other components increase cellular NEP activity (Ayoub and Melzig, 2006). In 2011 the small neuroprotective peptide humanin, whose cDNA was found in an AD patients brain (Hashimoto et al., 2001), was shown to increase NEP expression in the hippocampus of an AD mouse model (Niikura et al., 2011). Kynurenic acid (KYNA), one of the main products of the kynurenine pathway, is another neuroprotective component that is able to increase NEP expression, protein levels and activity in cultures of human neuroblastoma SH-SY5Y cells and mouse cortical neurons (Klein et al., 2013).

#### Summary

Several naturally occurring compounds, e.g., somastatin, estrogen, vitamin D, and components of green tea are able to upregulate NEP expression and/or activity. In contrast, oxidative stress was shown to lower the half-life and activity of the enzyme.

Most discussed in the last years is the regulation of NEP expression by AICD in a Notch-like signaling pathway.

## AICD Nuclear Signaling and Its Impact on the Regulation of NEP

### Striking similarities between Notch and APP processing

Like APP, the Notch receptors are single-pass type I transmembrane proteins that are processed with some striking similarities to APP (shown in Figure 1). In response to an extracellular signal, Notch is sequentially cleaved within its extracellular domain followed by intramembrane cleavage and the release of an extra- and intracellular fragment.

During maturation S1 cleavage of the Notch receptor precursor by a furin-like convertase takes place in the Golgi network and the fragments are shuttled to the cell surface as a non-covalently linked heterodimeric receptor molecule (Kopan and Turner, 1996; Logeat et al., 1998). The extracellular domain of Notch contains epidermal growth factor (EGF)-like repeats which mediate the binding of DSL ligands (Delta, Serrate/Jagged). This initiates S2 cleavage of Notch catalyzed by a member of the ADAM/tumor necrosis factor-α-converting enzyme (TACE) metalloprotease family releasing the Notch extracellular domain and the transient intermediate peptide Notch extracellular truncation (NEXT) (Mumm et al., 2000). In contrast to Notch processing there is no equivalent APP maturation and ligand binding necessary for APP to be further processed. Like the APP CTFs, NEXT is a substrate for the multimeric γ-secretase enzyme complex cleaving NEXT in its intramembrane region (De Strooper et al., 1999; Sastre et al., 2001). The γ-secretase mediated S3 cleavage of NEXT, which shares similarities with the γ-/ϵ-cleavage of APP CTFs regarding the membrane topology, presenilin-dependence, cleavage before a valine residue, and inhibition profile (Weidemann et al., 2002), releases the mobile cytoplasmic subunit Notch intracellular domain (NICD) from the membrane (Schroeter et al., 1998). NICD migrates to the nucleus and heterodimerizes with CSL (CBF1-SU(H)-LAG1) (Schroeter et al., 1998), which represses Notch target genes through recruitment of corepressor complexes in the absence of AICD. NICD displaces these corepressors, recruits a coactivator complex, and hence activates transcription. A role of NICD in the transcriptional regulation of several genes is well established (Borggrefe and Oswald, 2009). Due to the obvious similarities between APP and Notch in their protein structure and proteolytic cleavage, it is reasonable to assume that APP takes part in the regulation of other genes via its intracellular domain as well. Such a function of AICD is controversially discussed.

#### Summary

The high similarities between APP and Notch receptor processing by ADAM and γ-secretase activity leading to the generation of a large ectodomain and a short intracellular fragment indicate that AICD is likely to regulate the expression of multiple genes similar to NICD.

### The mechanisms of AICD nuclear signaling

**Figure 3:**
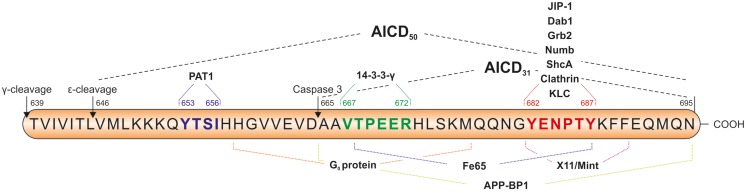
**Sequence of the APP intracellular domain (AICD) including important sequence motifs and adapter protein binding sites**. The YTSI sequence is required for basolateral sorting of APP, the VTPEER site is involved in pathophysiology and includes the Thr668 residue, which is the major phosphorylation site of APP. The GYENPTY sequence is reported to be important for the interaction of AICD with adaptor proteins containing a phosphotyrosine-binding (PTB) domain. Caspase-3 cleavage of AICD takes place between D664 and A665 and results in the formation of C31 peptide. The amino acid numbers are referring to the APP695 isoform.

Within AICD there are several sequence motifs of functional relevance which are summarized in Figure 3, e.g., the ^653^YTSI sequence, the ^667^VTPEER sequence, and the ^681^GYENPTY motif with the aa numbers referring to the APP695 isoform. Tyr653 in the YTSI sequence is required for basolateral sorting of APP in polarized MDCK cells (Lai et al., 1998). The VTPEER site appears to be involved in pathophysiology and Thr668 is the major phosphorylation site of APP (Suzuki and Nakaya, 2008). Far more attention has been addressed to the GYENPTY sequence which includes a NPXY motif serving as internalization signal for membrane proteins (Chen et al., 1990) and interacting with adapter proteins containing a phosphotyrosine-binding (PTB) domain (Uhlik et al., 2005). Interestingly, the residue Tyr682 is essential for the function of APP in developmental processes. Barbagallo et al. (2011) showed expression of APP with the Y682G mutation in APLP2-KO mice not to be able to compensate the early postnatal lethality and neuromuscular synapse defects of APP/APLP2-KO mice.

**Figure 4:**
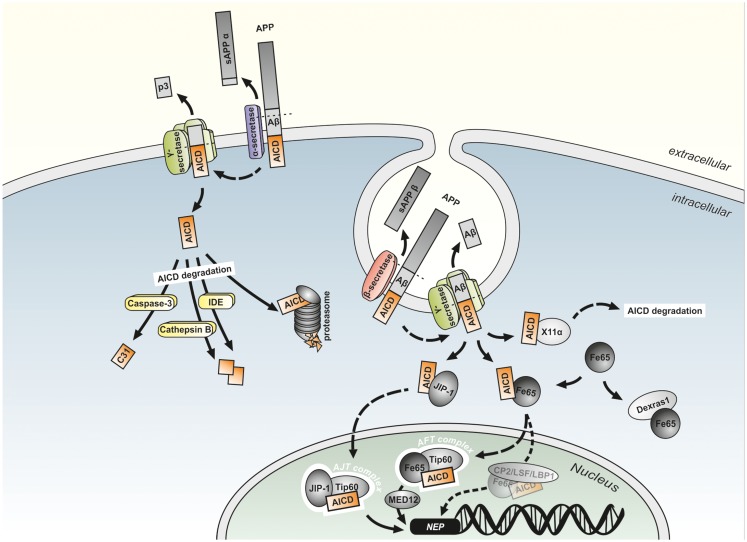
**Model of potential mechanism of AICD-mediated gene regulation**. The two different APP cleavage pathways have been shown to occur in distinct subcellular localizations. While the non-amyloidogenic pathway by α- and γ-secretase cleavage takes place at the plasma membrane, the amyloidogenic APP processing is discussed to take mainly place in endosomes. The APP intracellular domain (AICD) generated by non-amyloidogenic APP processing is rapidly degraded by, e.g., the proteasome, insulin-degrading enzyme (IDE), Cathepsin B, and Caspase-3 into smaller fragments. In contrast, AICD generated by amyloidogenic APP processing can be stabilized by binding to Fe65 or JIP-1 and translocated to the nucleus, where the gene regulatory AFT (AICD, Fe65, Tip60) or AJT (AICD, JIP-1, Tip60) complexes are formed. Alternatively to Tip60, the transcription factor CP2/LSF/LBP1 is hypothesized to interact with Fe65 and activate AICD-mediated gene transcription. Binding to the MED12 protein links these complexes to the RNA polymerase transcription apparatus. Additionally binding of AICD to MINT1/X11α or the interaction of Dexras1 with the PTB2 domain of Fe65 results in an inhibition of Fe65/AICD induced transcription.

After first description of AICD (then termed AID) by Passer et al. (2000), early experiments regarding a potential function of AICD in gene transcription were carried out by Cao and Sudhof in 2001 by using fusion proteins of APP and the DNA binding domains of the transcription factors Gal4 and LexA in combination with Gal4 and LexA dependent reporter plasmids to measure transactivation of transcription. Coexpression of the fusion proteins with the reporter plasmids resulted in only minor transactivation of transcription in HEK293, COS7, and HeLa cells, indicating that APP may need binding of a cofactor. Fe65 was identified as the major protein interacting with the APP cytoplasmic tail by yeast two-hybrid screens. Indeed, cotransfection of Fe65 and the APP-Gal4 or APP-LexA fusion proteins greatly stimulated transcription showing Fe65 to be the cofactor required for the function of AICD in gene regulation. Further yeast two-hybrid assays revealed an interaction of Tip60 with the PTB1 site of Fe65. GST-Tip60 efficiently pulled down APP together with Fe65 indicating the existence of a stable trimeric complex composed of the APP cytoplasmic tail, Fe65 and Tip60 that is able to transactivate transcription *in vitro* (Cao and Sudhof, 2001). The members of the Fe65 protein family, Fe65 and the Fe65 like proteins 1 and 2 (Fe65L1 and Fe65L2) are multidomain adaptor proteins that form multiprotein complexes. They all have a WW domain and two PTB domains and bind to AICD via their PTB2 domain (McLoughlin and Miller, 2008). Fe65 increases the stability of AICD (Kimberly et al., 2001; Kinoshita et al., 2002), which has a reported half-life of not more than 5 min (Cupers et al., 2001) due to its rapid degradation in the cytosol by IDE (Edbauer et al., 2002; Farris et al., 2003), the proteasome (Nunan et al., 2003), and the endosomal/lysosomal protease Cathepsin B (Vingtdeux et al., 2007; Asai et al., 2011) enabling AICD to translocate to the nucleus (see Figure 4). Further, the importance of Fe65 in APP function *in vivo* is indicated by the phenotype of Fe65/Fe65L1 gene deleted mice, showing cortical dysplasia like APP/APLP1/APLP2 triple KO animals (Guenette et al., 2006). Tip60 is a histone acetyltransferase (Yamamoto and Horikoshi, 1997) acting in chromatin remodeling, DNA repair, transcription, and apoptosis (Ikura et al., 2000). The existence of the trimeric protein complex consisting of fluorescent protein-tagged AICD, Fe65, and Tip60 (termed AFT-complex) was further confirmed by colocalization of the three proteins in spherical nuclear spots (Von Rotz et al., 2004; Goodger et al., 2009). Moreover, Konietzko et al. (2010) demonstrated by confocal analysis that nuclear export blockade allows to reveal the nuclear localization of endogenous AICD at the level of nuclear transcription territories.

All these observations resulted in the model that Fe65 binds AICD after its release from the membrane by γ-secretase cleavage, followed by the translocation of the Fe65/AICD complex to the nucleus, where it associates with Tip60 leading to the formation of AFT complexes. Although this model is suggested by most authors, there are some studies suggesting alternative mechanisms for signal transduction by APP (Muller et al., 2008). These models suggest an independent translocation of AICD and Fe65 to the nucleus (Nakaya and Suzuki, 2006), a conformational change of Fe65 induced by AICD enabling it to translocate to the nucleus where it binds Tip60 to form a transcriptionally active AFT complex (Cao and Sudhof, 2004), or the recruitment of Tip60 through Fe65 by the APP C-terminus independently from γ-secretase cleavage that results in the phosphorylation, stabilization and activation of Tip60 by cyclin-dependent kinase (CDK) leading to the translocation of the Tip60/Fe65 complex into the nucleus (Hass and Yankner, 2005). Arguing against some of these alternative models, the translocation of AICD to the nuclear compartment was reported by several studies. AICD was found to be located in the nuclei of cultured cells and even in cortices of APP transgenic mice (Chang et al., 2006).

Alternatively to Tip60, the transcription factor CP2/LSF/LBP1 can interact with Fe65 and bind to the promoter region of potential AICD target genes (Zambrano et al., 1998; Kim et al., 2003). MED12 protein, which is part of the Mediator complex, a large protein complex transducing signals from specific transcription factors to RNA polymerase II, binds to Fe65 and Tip60 in the presence of AICD, providing a direct link of the AICD/Fe65/Tip60 complex to the RNA polymerase II general transcription apparatus (Xu et al., 2011).

More than 20 other adaptor proteins that bind AICD or the Fe65-AICD complex have been identified (Borquez and Gonzalez-Billault, 2012) (see Figure 3), the most prominent examples are reviewed here. Nuclear signaling of APP is prevented by MINT-1/X11α by retaining AICD in the cytosol instead of translocating it to the nucleus (Von Rotz et al., 2004). The protein Dexras1 was shown to bind to the PTB2 domain of Fe65 and to compete with APP for binding to Fe65 leading to a suppression of FE65-APP mediated transcription (Lau et al., 2008). Interaction of AICD with the ubiquitin-like Nedd8 results in the dissociation of AICD from Fe65 combined with the reduction of AFT complexes and of AICD transcriptional activity (Lee et al., 2008). In contrast, the Janus kinase interacting protein-1 (JIP-1) in combination with AICD can activate gene expression in a Fe65 independent manner (Scheinfeld et al., 2003), with transport of AICD to nuclei and the subsequent docking to Tip60 resulting in the formation of AICD-JIP-1-Tip60 (AJT) complexes showing a different, speckle like morphology compared to AFT complexes (Von Rotz et al., 2004). Dimeric 14-3-3γ binds to the VTPEER sequence of AICD and to a sequence between the WW domain and the first PTB domain of Fe65 and facilitates gene transactivation by enhancing the AICD-Fe65-association (Sumioka et al., 2005). The phosphorylation status of AICD affects the affinity of binding partners and therefore influences the Fe65/AICD-mediated transcriptional control (Ando et al., 2001).

Experimental evidence suggests that not all pools of AICD are active in nuclear signaling. As Aβ, sAPPβ and AICD are mainly formed from APP695, this isoform mainly expressed in neurons seems to have a higher affinity or turnover by β-secretase compared to the other isoforms. In line, APP751 and APP770 were shown to be mainly processed by α- and γ-secretase cleavage (Kametani et al., 1993; Belyaev et al., 2010). Only the overexpression of APP695 results in an increase of AICD levels in nuclear fractions of SH-SY5Y cells (Belyaev et al., 2010). This may indicate a neuronal specificity of the AICD-mediated gene regulatory mechanism with APP695 being the major APP isoform in neuronal, but not in non-neuronal cells (Sandbrink et al., 1994). Although AICD derives from both amyloidogenic and non-amyloidogenic APP processing pathways and an α-secretase-dependent AICD generation was reported (Kume et al., 2004), several studies demonstrated the β-secretase mediated APP cleavage in endosomal compartments to be mainly responsible for the generation of transcriptionally active AICD species. Interestingly, Passer et al. (2000) already reported in the first study describing the existence of AICD, that overexpression of C99 results in larger amounts of AICD than overexpression of C83. Blocking the amyloidogenic APP processing by inhibition of endocytosis or inhibition/genetic deletion of BACE1 in fibroblasts, HEK293 cells, and primary neurons leads to a reduced translocation of AICD to nuclear AFT complexes, while inhibition of α-secretase has no effect on AFT-complex generation (Goodger et al., 2009). This was confirmed by Belyaev et al. (2010) showing that inhibition of β- and γ-, but not α-secretase in SH-SY5Y cells leads to abolished AICD-mediated upregulation of the AICD target gene NEP. The results of the studies mentioned above resulted in the model (illustrated in Figure 4), that AICD released by α/γ-secretase cleavage at the plasma membrane is rapidly degraded in the cytosol by IDE and other proteases like Cathepsin B and truncated into C31 by caspase-3 activity. In contrast, the AICD generated by amyloidogenic APP processing requiring the endocytotic pathway, can translocate to the nucleus due to a shorter distance resulting in less degradation of the peptide. There it forms a complex with MED12, Fe65, and Tip60 that is able to regulate gene expression (Beckett et al., 2012). Conversely, in a study by Flammang et al. AICD was shown to be predominantly derived from C99. In this study C84 or C100 was overexpressed in *E. coli*, purified and supplemented with mammalian γ-secretase containing membranes. Analyzing the generated amount of AICD revealed that under these conditions γ-secretase has a higher turnover of C99 compared to C83 resulting in a higher level of C99 derived AICD. Similar results were obtained by utilizing cell-derived, membrane-embedded C83/C99 from C83/C99 overexpressing mammalian cells (Flammang et al., 2012). This is consistent with the data generated by Passer et al., Goodger et al., and Belyaev et al., but argues against the cytoplasmatic AICD degradation to be responsible for the inefficiency of C83 derived AICD in forming AFT complexes and regulating transcription. The existence of a substrate inhibitory domain in C83 (ASID, Aβ17–23) that inhibits γ-secretase activity is a possible explanation for the reduced affinity of γ-secretase toward C83 (Tian et al., 2010).

#### Summary

Amyloid precursor protein intracellular domain binds to Fe65 after its release from the membrane. This is followed by the translocation of the AICD/Fe65 complex to the nucleus, where the association with the histone acetyltransferase Tip60 and the formation of the transcriptionally active AFT complex takes place. Experimental evidence suggests that the AICD active in nuclear signaling is produced mainly from the APP695 isoform in a β-/γ-secretase-dependent manner.

### Impact of AICD in the regulation of NEP

Remarkably, in the case of an AICD-mediated upregulation of NEP, one APP cleavage product, AICD indirectly regulates the lifetime of another APP cleavage product, Aβ, by upregulation of its degradation resulting in a regulatory cycle, in which γ-secretase is responsible for Aβ production and modulates its degradation at the same time. Importantly, it has been shown, that Aβ and AICD generation by γ-secretase could be independently regulated (Chen et al., 2006; Wiley et al., 2007; He et al., 2010). In this cycle decreased AICD formation, for example by reduction of γ-secretase activity, genetic deletion of APP, or prevention of AICD transport to the nucleus, should result in reduced NEP expression leading to higher Aβ levels. This has been analyzed by several groups over the past years with inconsistent outcomes. An overview of the performed experiments and the generated results is given in Table 1.

**Table 1:** **Summary of studies elucidating the link between AICD and NEP (↑ increased, ↓ decreased, Δ genetic deletion)**.

Study	Used cells	Used mouse model	NEP expression (% of control)	NEP level (% of control)	NEP activity (% of control)	Rescue?	Comments
Pardossi-Piquard et al. (2005)	MEF ΔPS1ΔPS2		↓20% (approximately)	↓29%	↓17.25% (homogenate) ↓14.81% (intact cells)	By transfection of PS1, PS2, PS1 + PS2, and AICD50, AICD59; higher effect by cotransfection of AICD50/59+ Fe65+ Tip60	No effect of ΔPS1 or ΔPS2
Hebert et al. (2006)	MEF ΔPS1ΔPS2			No effect			
Chen and Selkoe (2007)	MEF ΔPS1ΔPS2			↓		Transfection of PS1 has no effect	NEP level reduced in ΔPS2, but increased in ΔPS1
Pardossi-Piquard et al. (2005)	BD8 ΔPS1ΔPS2			↓51%	↓72.23% (homogenate)	By transfection of AICD50 and AICD59	
					↓52.27% (intact cells)	
Chen and Selkoe (2007)	BD8 ΔPS1ΔPS2			No effect (modest effect in cells harvested in Tris 0.5%Triton buffer)		Transfection of PS1, PS2, or cotransfection of AICD60+ Fe65+ Tip60 has no effect	
Huysseune et al. (2009)	MEFΔPS1ΔPS2		No effect				Expression measured by microarray
Pardossi-Piquard et al. (2005)		ΔPS1ΔPS2 mouse brain		↓59%	↓71%		No effect of ΔPS1
Hebert et al. (2006)		ΔPS1 mouse embryo brain (E14,5)		No effect			
Hebert et al. (2006)	MEF ΔAph1a			No effect			
Hebert et al. (2006)		ΔAph1a whole mouse embryo (E9,5)		No effect			
Pardossi-Piquard et al. (2006)	MEF ΔNCT		↓	↓	↓Homogenates	By expression of NCT	
					↓Intact cells	
Pardossi-Piquard et al. (2005)	Inhibition of γ-secretase in MEF WT				↓ 50% (intact cells) by DAPT, ↓ by other γ-secretase-inhibitors		Used γ-secretase-inhibitors: DAPT, DFK167, L 685,458; used concentration for DAPT: 2 μM, 48 h
Pardossi-Piquard et al. (2005)	Inhibition of γ-secretase in TSM1 neurons				↓ 69.6% (intact cells) by DFK167		
Pardossi-Piquard et al. (2005)	Inhibition of γ-secretase in primary cultured neurons				↓ 43.7% (intact cells) by DFK167		
Hebert et al. (2006)	Inhibition of γ-secretase in MEF WT, Hela WT, cos7 WT, and N2a WT			No effect			Use of γ-secretase inhibitor X and DAPT: 10 μM, 16–18 h
Chen and Selkoe (2007)	Inhibition of γ-secretase in BD8 WT and HEK293T WT cells			No effect			Use of γ-secretase inhibitor Compound E
Chen and Selkoe (2007)	Inhibition of γ-secretase in MEF WT			No effect			Use of γ-secretase inhibitor DAPT: 500 nM, 48 h
Xu et al. (2011)	Inhibition of γ-secretase in NB7 cells and SK-N-SH cells		↓				Use of γ-secretase inhibitor DAPT: 25 and 50 μM, 48 h
Pardossi-Piquard et al. (2005)	MEF ΔAPPΔAPLP2		↓ 70%	↓ 8%	↓ 20% (homogenate) ↓ 13% (intact cells)	By expression of APP in ΔAPP, by expression of APLP2 in ΔAPLP2, and by expression of ALID1 or ALID2 or AICD50 (Luciferase assay) in ΔAPPΔAPLP2	Also effects in ΔAPP and ΔAPLP2
Hebert et al. (2006)	MEF ΔAPPΔAPLP2			No effect			
Huysseune et al. (2009)	MEFΔAPP		No effect	No effect			Expression measured by microarray
Huysseune et al. (2009)	MEFΔAPPΔAPLP2			No effect			
Belyaev et al. (2010)	APP695 overexpression in SH-SY5Y cells		↑(sixfold)				Effect reduced after treatment with γ-secterase inhibitor L685,458
Belyaev et al. (2009)	APP knockdown in NB7 cells		↓				
Xu et al. (2011)	APP knockdown in NB7 and SK-N-SH cells		↓				
Pardossi-Piquard et al. (2005)		ΔAPP mouse brain			↓53%		
Pardossi-Piquard et al. (2005)		ΔAPPΔAPLP2 mouse brain			↓48%		Similar effect in ΔAPPΔAPLP1
Hebert et al. (2006)		ΔAPPΔAPLP2 embryonic brain (E15,5)	No effect	Noeffect			Also no effect in ΔAPPΔAPLP1ΔAPLP2
Chen and Selkoe (2007)		ΔAPP mouse brain		No effect	No effect		
Chen and Selkoe (2007)		ΔAPLP2 mouse brain		No effect	No effect		
Pardossi-Piquard et al. (2005)	MEF Δp97Fe65		↓73%		↓48%		
Pardossi-Piquard et al. (2007)		Brains of AICD and Fe65 overexpressing mice		↑(control: +Fe65)			
Waldron et al. (2008)	Transfection of Fe65+ AICD accumulation in HEK293 cells		No effect				AICD accumulating is most likely generated from C83
Muller et al. (2007)	Induced expression of AICD and Fe65 in SHEP-SF cells		No effect				Expression measured by microarray
Xu et al. (2011)	MED12 knockdown in NB7 and SK-N-SH cells		↓				

Pardossi-Piquard et al. published, that γ-secretase inhibition and genetic deletion of PS1/PS2, nicastrin, Fe65, and the APP family members results in decreased NEP expression and activity *in vitro* and *in vivo.* In this studies NEP expression and activity were shown to be restored by transient expression of PS1, PS2, or the intracellular domains of APP, APLP1, and APLP2 (Pardossi-Piquard et al., 2005, 2006, 2007). In line with these findings, pharmacological inhibition of γ-secretase or RNAi-mediated APP knockdown in NB7 and SK-N-SH cells leads to significantly decreased NEP mRNA levels as reported by Xu et al. Interestingly, similar effects on NEP and other potential AICD target genes were observed by RNAi-mediated knockdown of MED12 identifying the protein as a transducer of AICD nuclear signaling for the first time (Xu et al., 2011). Furthermore, NEP gene promoters are transactivated by AICD in TSM1 neurons and fibroblasts shown by luciferase reporter assays (Pardossi-Piquard et al., 2005). A direct physical interaction of AICD with the NEP promoter was further confirmed by supergel shift assay analysis in HEK293 cells and chromatin immunoprecipitation (ChIP) (Pardossi-Piquard et al., 2005; Belyaev et al., 2009). AICD binds directly to the NEP promoter NB7 cells, which highly express NEP, and rat primary cortical neurons, but not in HUVEC (primary human umbilical vein endothelial) and SH-SY5Y cells, which only show a low NEP expression. In this study excess histone deacetylation was shown to be involved in NEP repression (Belyaev et al., 2009).

Other groups failed to reproduce the observed effects of AICD signaling on NEP expression, even by using broadly similar methodologies and partly the same cells for their experiments.

Hebert et al. (2006) found no consistent effect on NEP expression in several cell lines treated with γ-secretase-inhibitors and in fibroblasts bearing genetic deficiencies in the γ-secretase complex or the APP family members. There was also no alteration of NEP levels in tissues of APP/APLP1, PS1, or Aph1a gene disrupted mouse embryos. Chen et al. also reported the lack of both PS1 and PS2 expression in mouse embryonic stem cells (BD8) not to alter NEP levels. In PS double KO fibroblasts NEP levels are decreased, but rescued γ-secretase activity by introduction of PS1 failed to rescue the effect on NEP levels. Cellular NEP levels are unaffected as well by inhibition of γ-secretase in fibroblasts, BD8, and HEK293 cells and by introduction of AICD, Tip60, and Fe65 in PS double KO BD8, BD8 wildtype, and HEK293 wildtype cells. Moreover, there was no change in NEP levels and activity in APP and APLP2 single KO mouse brain homogenates found (Chen and Selkoe, 2007). In a further study, gene-chip microarray analysis revealed no alterations of NEP expression in APP KO and PS1/PS2 double KO fibroblasts along with no alterations of NEP protein levels in APP/APLP2 single and double KO fibroblasts (Huysseune et al., 2009).

Several factors influencing the experimental outcomes, for example cell types, clonal heterogeneity, density and age of the used cells, transgenic mouse models (single vs. double KO), and differences in incubation times/concentrations of the used inhibitors might explain the discussion about a function of AICD in gene regulation (Pardossi-Piquard et al., 2007; Bauer et al., 2011). Different cell lines may vary in the ratio of constitutive vs. regulated NEP expression. In consequence, detection of AICD regulated NEP expression is probably not possible in all cells. In line with this hypothesis Hong et al. (2012) failed to detect an influence of AICD on NEP in human prostate cells. As already mentioned, the distinct APP isoforms differ markedly in their ability to modulate NEP expression with transcriptionally active AICD being preferentially derived by β-secretase cleavage of APP695, the most prominent APP isoform in neuronal, but not in non-neuronal cells (Belyaev et al., 2010). This could additionally explain cell type specificity of the signaling pathway and the failure to observe AICD-mediated transcriptional activation in studies using non-neuronal cell lines.

Nevertheless, physical and functional interaction of AICD and regulatory elements of the NEP promoters has been clearly demonstrated (Pardossi-Piquard et al., 2005; Belyaev et al., 2009). Binding of AICD to these elements is followed by the displacement of HDAC1 (histone deacetylase 1) and hence the activation of transcription (Belyaev et al., 2009; Xu et al., 2011). The remodeling of chromatin by histone acetylation and deacetylation is an important mechanism of regulating gene expression in general. NEP expression in neuronal cells was shown to be repressed via competitive binding of HDACs to its promoter. Sodium valproate (valproic acid, VA), an anticonvulsant showing HDAC-inhibitory properties, is able to restore AICD promotor binding as well as NEP expression *in vitro* and *in vivo* and improves animal behavior and memory (Qing et al., 2008; Belyaev et al., 2009; Zhuravin et al., 2011; Nalivaeva et al., 2012), indicating the AICD dependent regulation of NEP to be an interesting therapeutical target.

Imatinib (Gleevec), a tyrosine kinase inhibitor used for the treatment of several cancers, shows another mode of action in the upregulation of NEP. Incubation of H4 cells with Imatinib results in reduced Aβ levels accompanied by increased levels of both AICD and NEP (Eisele et al., 2007). A causative link between these effects was later given by the observation that the Imatinib-induced NEP elevation is totally abolished by genetic deletion of APP/APLP2 in fibroblasts. The authors suggest, that Imatinib treatment slows down AICD degradation resulting in increased NEP levels, higher Aβ degradation and hence to a reduction in Aβ levels (Bauer et al., 2011).

#### Summary

The impact of AICD on NEP expression has been demonstrated in several models by the use of different methods. In line a direct interaction between AICD and the NEP promoter region has been shown. In contrast, other studies exist reporting no AICD-mediated NEP regulation. NEP expression is upregulated by sodium valproate and Imatinib via inhibition of HDACs and of AICD degradation, respectively.

In conclusion, the regulation of NEP by AICD cannot be assumed in general, but rather for specific pools of AICD in certain tissues. It represents a potential therapeutic target for AD. However, further studies elucidating potential side effects are necessary, especially by keeping in mind the high repertoire of NEP substrates and the potential role of AICD in the regulation of genes involved in a variety of cellular functions, as for example the initiation of apoptosis. A similar situation occurs by the approach of γ-secretase inhibition to treat AD because of various known targets of the γ-secretase.

### Further potential AICD target genes

The controversial results obtained by AICD-mediated regulation of NEP is also reflected by other targets assumed to be regulated by AICD. Here we briefly summarize these studies to further evaluate the general transcriptional regulation mediated by AICD and therefore to get an impression why different results might have been obtained in literature.

Potential target genes of AICD nuclear signaling include retinoic acid-responsive genes (Gao and Pimplikar, 2001), KAI1/CD82 (Baek et al., 2002), glycogen synthase kinase-3β (GSK3β) (Kim et al., 2003), APP, BACE1, Tip60 (Von Rotz et al., 2004), NEP (Pardossi-Piquard et al., 2005), p53 (Alves da Costa et al., 2006), Fibronectin1 (FN1), α2-actin, transgelin (SM22), tropomyosin 1 (TPM1), flavoprotein oxidoreductase MICAL2 (MICAL2), Ras-associated protein (RAB3B) (Muller et al., 2007), EGF receptor (EGFR) (Zhang et al., 2007), LRP (LRP1) (Liu et al., 2007), Cyclins B1 and D1 (Ahn et al., 2008), vesicular glutamate transporter (VGLUT2) (Schrenk-Siemens et al., 2008), C/EBP homologous protein (CHOP) (Takahashi et al., 2009), Aquaporin 1 (Huysseune et al., 2009), S100a9 (Ha et al., 2010), ApoJ/clusterin (Kogel et al., 2012), patched homolog 1 (Ptch1), transient receptor potential cation channel subfamily C member 5 (TRPC5) (Das et al., 2011), serine-palmitoyl transferase (SPT) subunit SPTLC2 (Grimm et al., 2011a), alkyl-dihydroxyacetonephosphate-synthase (AGPS) (Grimm et al., 2011b), GD3 synthase (GD3S) (Grimm et al., 2012), Stathmin1 (Muller et al., 2013), and PGC1α (Robinson et al., 2013). A summary of these genes including the experimental procedures to investigate a potential impact of AICD on their expression are listed in Table 2.

**Table 2:** **Genes discussed to be regulated by AICD (↑ increased, ↓ decreased)**.

AICD target gene	Physiological functions	Regulation	Experimental design	Study
Retinoic acid-responsive genes	Cell–cell communication, cell development	↓	Luciferase assay in CV1 cells: plasmid-dose-dependent repression by C59, little repression by C57	Gao and Pimplikar (2001)
KAI1/CD82	Suppression of tumor metastasis	↑	Recruitment of the AFT to the KAI1 promotor displaces N-CoR/TAB2/HDAC3 corepressor-complex in absence of interleukin-β in APP transgenic mice	Baek et al. (2002)
		↑	Inducible AICD overexpression in HEK293 cells leads to upregulation of KAI1 mRNA levels	Von Rotz et al. (2004)
Glycogen synthase kinase-3β (GSK3β)	Regulation of cell cycle, cell proliferation, apoptosis; glycogen metabolism	↑	AICD57, AICD59, and C31 induce expression of GSK3β in PC12 cells and rat primary cortical neurons	Kim et al. (2003) Ryan and Pimplikar (2005), Von Rotz et al. (2004)
		↑activity	Stimulation of GSK3β activity in AICD transgenic mice, no change in mRNA and protein levels of GSK3β	
		↑	Inducible AICD overexpression in HEK293 cells leads to upregulation of GSK3β mRNA levels	
Amyloid precursor protein (APP)	Cell adhesion, synaptogenesis, modulation of synaptic plasticity, neurite outgrowth, neuronal migration	↑	Inducible AICD overexpression in HEK293 cells leads to upregulation of APP mRNA levels	Von Rotz et al. (2004)
β-site APP cleaving enzyme 1 (BACE1)	Amyloidogenic APP cleavage	↑	Inducible AICD overexpression in HEK293 cells leads to upregulation of BACE1 mRNA levels	Von Rotz et al. (2004)
Tip60	Histone acyltransferase, chromatin remodeling, DNA repair, transcription and apoptosis	↑	Inducible AICD overexpression in HEK293 cells leads to upregulation of Tip60 mRNA levels	Von Rotz et al. (2004)
Neprilysin (NEP)	Aβ degradation, neuropeptide signaling, regulation of vascular tone	↑	For detailed list of studies and experimental design see Table 1	see Table 1
p53	Tumor suppression, apoptosis	↑	Deficient γ-secretase activity and APP/APLP2 depletion reduce expression and activity of p53 while AICD overexpression increases p53 activity, transactivation of murine and human p53 promoters in wildtype blastocysts, PS-deficient blastocysts, and HEK293 cells	Alves da Costa et al. (2006)
Fibronectin1 (FN1); α2-Actin; transgelin (TAGLN, SM22); tropomyosin 1 (TPM1); flavoprotein oxidoreductase MICAL2 (MICAL2); Ras-associated protein (RAB3B)	Organization and dynamics of the cytoskeleton	↑	Expression of target genes analyzed by microarray and RT-PCR in Tet21 cells (derived from SHEP-SF) after induction of AICD alone or AICD in combination with Fe65	Muller et al. (2007)
Epidermal growth factor receptor receptor (EGFR)	Cell cycle, cell proliferation, and differentiation	↓	Fibroblasts deficient for γ-secretase activity and APP show increase of EGFR, direct binding of endogenous AICD to the EGFR promoter	Zhang et al. (2007)
Lipoprotein receptor-related protein-1 (LRP1)	Brain apolipoprotein E and cholesterol metabolism, signal reception	↓	Enhanced expression and function of LRP1 by deletion of APP and APLP2 or components of the γ-secretase complex that is reversed by expression of AICD, AFT complex interacts with LRP1 promoter and suppresses transcription	Liu et al. (2007)
Cyclins B1 and D1	Regulation of cell cycle	↑	Increased levels of cyclin B1 and cyclin D1 in differentiated PC12 cells or rat primary cortical neurons expressing APPswe or AICD	Ahn et al. (2008)
Vesicular glutamate transporter 2 (VGLUT2)	Neurotransmission, transport of glutamate	↑	Decreased expression of VGLUT2 in glutamatergic neurons differentiated from mouse embryonic stem cells lacking APP and APLP2 genes, effects restored by expression of AICD; similar decrease of VGLUT2 expression by blocking γ-secretase cleavage of APP in wt neurons	Schrenk-Siemens et al. (2008)
C/EBP homologous protein (CHOP)	ER-stress, unfold protein response, apoptosis	↑	CHOP levels are increased by APP or AICD overexpression, attenuated by treatment with a γ-secretase inhibitor; APP knockdown attenuated cell death and CHOP upregulation; direct association of AICD with the CHOP promoter	Takahashi et al. (2009)
Aquaporin 1 (AQP1)	Water channel	↑	AQP1 expression decreased in MEF lacking APP or PS, AQP1 expression was restored by stable expression of APP or PS2 but not by APP lacking the intracellular C-terminal domain	Huysseune et al. (2009)
S100a9	Calcium-binding, inflammation	↑	Upregulation of S100a9 in brains of APP C-terminus transgenic mice; transfection of BV2 microglia cells with APP CT50 or CT99 leads to increased S100a9 mRNA level, effects further examined in luciferase reporter assays	Ha et al. (2010)
ApoJ/clusterin	Lipoprotein, transport of lipids	↓	AICD down-regulates mRNA levels of ApoJ/clusterin	Kogel et al. (2012)
Transient receptor potential cation channel subfamily C member 5 (TRPC5)	Ion channel	↓	Decrease in TRPC5 mRNA and protein levels in mouse N2a cells overexpressing AICD	Das et al. (2011)
Patched homolog 1 (Ptch1)	Brain development, cell proliferation and division, sonic hedgehog signaling	↑	Increase in Ptch1 mRNA and protein levels in mouse N2a cells overexpressing AICD	Das et al. (2011)
Alkyl-dihydroxyacetone phosphate-synthase (AGPS)	Plasmalogen synthesis	↑	AGPS mRNA levels reduced in MEF deficient for PS1 and PS2, APP and APLP2 or the APP C-terminus, effects on AGPD also observed in APP^−/−^, APP^±^, APP^±^APLP^−/−^, and APPC-terminus deficient mouse brains and in human SH-SY5Y Fe65-knockdown cells	Grimm et al. (2011b)
Serine-palmitoyl transferase subunit SPTLC2	Sphingolipid synthesis	↓	SPTLC2 levels increased in PS1/PS2-deficient, APP/APLP2-deficient MEF and MEF lacking the C-terminus of APP; incubation with a synthetic AICD peptide decreases SPTLC2 expression; Fe65-knockdown SH-SY5Y cells increases SPTLC2 expression, higher SPTLC2 mRNA level in the brain of mice lacking APP, or the APP C-terminus	Grimm et al. (2011a)
GD3 synthase (GD3S)	Ganglioside synthesis	↓	GD3S levels and activity elevated in MEF deficient for PS1/PS2, APP/APLP2, or the APP C-terminus, rescue of the effect by incubation with synthetic AICD peptide, Fe65-knockdown SH-SY5Y cells increases GD3S expression; level of brain GD3S increased in APP knockout mice and mice expressing an APP deletion construct lacking the C-terminal region	Grimm et al. (2012)
Stathmin1	Regulation of microtubule dynamics	↓	AFT expressing cells show downregulation of stathmin1, validated by mass-spectrometry; opposite regulation of stathmin1 in cells lacking all three members of the APP family	Muller et al. (2013)
Peroxisome proliferator-activated receptor-γ coactivator 1α (PGC1α)	Transcriptional coactivator, regulation of mitochondrial biogenesis, and energy metabolism	↑	Reduced PGC1α expression in MEFPS1^−/−^ΔPS2^−/−^, in MEFAPP^−/−^APLP2^−/−^, in MEF deficient for the APP C-terminus and in SH-SY5Y FE65-knockdown cells; effects could be reversed by AICD treatment; PGC1α mRNA level also reduced in APP KO mice, and mice deficient for the APP C-terminus	Robinson et al. (2013)

The metastasis suppressor KAI1 was the first identified functionally significant AICD target gene. A direct interaction of the AFT complex with the KAI1 promoter region initiates the displacement of the N-CoR/TAB2/HDAC3 corepressor complex and leads to an increase of KAI1 mRNA and protein levels in the central nervous system of APP transgenic mice. In these mice the protein levels of APP, Fe65 and Tip60 are dramatically increased as well (Baek et al., 2002). The expression of glycogen synthase kinase-3β (GSK3β), which is implicated in the hyperphosphorylation of tau in AD, was also shown to be upregulated by AICD. Overexpression of AICD in PC12 cells and in rat primary cortical neurons induces the expression of GSK3β and its promoter activity. In this study the enhanced expression of GSK3β is followed by an increase in tau phosphorylation and a reduction of β-catenin levels, leading to apoptosis (Kim et al., 2003). The GSK3β mediated tau phosphorylation induced by AICD may provide a link between APP and tau, the two proteins that are responsible for the major pathologic hallmarks of AD. Von Rotz et al. (2004) confirmed the upregulation of KAI1, APP, BACE1, Tip60, and GSK3β, but not Fe65, in HEK293 cells after inducible AICD overexpression, indicating a feed-forward mechanism, in which AICD upregulates the expression of its own precursor APP, the APP cleaving enzyme BACE1, involved in its generation, and Tip60, involved in its signaling pathway. The elevation of GSK3β activity by AICD was validated *in vivo* in AICD transgenic mice, where it results in enhanced phosphorylation of CPMP2 protein, a GSK3β substrate involved in axonal guidance (Ryan and Pimplikar, 2005). AICD is further linked to apoptosis by the observation that the proapoptotic tumor suppressor gene p53 is affected by AICD nuclear signaling. This may be an additional explanation for the AICD induced and Tip60 dependent cell death, which is abolished by p53 deficiency (Passer et al., 2000; Kinoshita et al., 2002; Ozaki et al., 2006). Genetic depletion of APP/APLP2 or PS and reduction of γ-secretase activity leads to decreased expression and activity of p53 and in line with this to a reduced activation of the p53 promoter. p53 expression is also diminished *in vivo* in the brains of PS- or APP-deficient mice. In contrast, enhanced p53 activity and p53 promoter transactivation was observed in AICD overexpressing blastocysts and HEK293 cells (Alves da Costa et al., 2006).

In contrast to the studies mentioned above, several authors failed to observe an impact of AICD on the regulation of the same target genes. Hébert et al. reported the protein levels of the potential AICD target genes APP and GSK3β not to be altered in cells treated with γ-secretase-inhibitors or in biological models genetically deficient for AICD generation. In line, analysis of the transactivation properties of AICD on the promoter regions of KAI1 and APP only showed modest effects (Hebert et al., 2006). Transcriptome analysis of a human neuroblastoma cell line inducible for expression of AICD, Fe65 or both failed to identify differential expression of KAI1, GSK3β, APP, and NEP. In this study real-time quantitative PCR confirmed that AICD or AICD/Fe65 expression is not associated with changes in the expression of either KAI1 or GSK3β (Muller et al., 2007). In a study by Waldron et al. increased AICD levels generated in the presence of NH4Cl, failed to stimulate the APP and KAI1 promoter regions in luciferase reporter assays. The lack of a transactivation potential of AICD on these genes is further demonstrated by the unchanged NEP, KAI1, BACE1, EGFR, Tip60, and p53 mRNA levels. It should be noted, that AICD accumulating under these conditions is derived from C83, indicating that it represents an AICD species that is discussed not to be transcriptionally active as mentioned above (Waldron et al., 2008).

#### Summary

Many conflicting data and models concerning the role of AICD in promoting the expression of several potential AICD target genes including NEP have emerged. This might be due to the short half-life of AICD, which *per se* complicates the finding of experimental conditions to elucidate the physiological role of AICD.

### Analysis of the impact of AICD in gene regulation: Experimental challenges and further approaches

Up to now the impact of AICD in gene regulation in general and in transcriptional regulation of NEP in particular is highly controversially discussed. As reviewed above several controversial findings have been published, which might be due the extraordinary experimental challenges of this topic. In this paragraph we will summarize these challenges and suitable methods to deal with these problems, briefly explaining the advantage and disadvantage of each method.

Addressing the role of AICD in gene regulation, the most prominent problem to deal with is the extremely short half-life of the peptide (Cupers et al., 2001) caused by proteolytic degradation by caspases, IDE or the proteasome (Gervais et al., 1999; Edbauer et al., 2002; Farris et al., 2003; Nunan et al., 2003). Especially the use of non-overexpressing systems or incubation of cells with physiological concentrations of AICD results in a false negative study outcome. However, it is possible to inhibit the AICD degrading processes with specific inhibitors, such as MG-132 and Epoxomicin for proteasome (Gersbacher et al., 2013) or Bacitracin for IDE resulting in an increased half-life of AICD. Using such inhibitors one has to be aware of the partially high cytotoxicity and that beside AICD the degradation of a huge amount of other proteins and peptides is also inhibited, making the conclusion from the obtained results more ambiguous.

Alternatively, higher concentrations of AICD can be used for incubation. However, it has to be taken into consideration that artificial assembly of AICD with adapter proteins might be the consequence, generating false positive results. The same problem occurs with APP overexpressing cell lines, where additionally missorting and protein accumulation might take place. Inducible promotors like used in TET-systems could help to overcome these challenges. Moreover, enhancing the AICD uptake by using lipofection reagents might be beneficial.

Beside the addition of the peptide and expressing AICD or its precursor constructs, reasonable results could be obtained by reducing the cellular amount of AICD. In principle APP KO, PS KO, and BACE1 KO cells are available and often used for analysis. Utilizing these KOs, it has to be mentioned that beside AICD the generation of further APP cleavage products is inhibited as well, making it impossible to differentiate between the impact of AICD in comparison to other APP derived fragments.

Moreover, APP might accumulate due to inhibiting its catabolism and it has already been shown that APP itself is involved in transcriptional regulation as summarized in the next section. Beside the lack of other APP cleavage products and the accumulation of APP, γ-secretase as well as BACE1 have several other substrates, which are also affected by the KO, making it necessary to combine different approaches and to look for intersections of the obtained results. In addition to the false positive results obtained by affecting other proteins and mechanisms, APP KO models might also lead to false negative findings. As mentioned in Section “Proteolytic Processing of APP” not only APP but the whole APP family has AICD like domains (ALID1/2 in the case of APLP1 and APLP2), which can be released and are potentially transcriptionally active. It is assumed that ALID1 and ALID2 are at least partially able to compensate the lack of AICD. Therefore it should be considered to utilize combined APP/APLP1/APLP2-KO cells to investigate the effects of AICD. Furthermore it has to be pointed out that mostly, because of their ability to immortalize, mouse embryonic fibroblasts (MEFS) are used. It has been shown that AICD transcriptional regulation is at least in some cases tissue specific. Therefore results obtained from fibroblasts cannot be automatically transferred to other tissues like brain. To avoid these problems knock downs of the relevant proteins in neuronal cell lines like N2A (murine) and SH-SY5Y (human) cells or pharmacological inhibition of the secretases in these systems can be additionally used. However, it has to be balanced whether the toxicity of the inhibitors and the incomplete knock down of the proteins might affect the obtained findings. Moreover, beside the results found in cell culture experiments *in vivo* relevance has to be proofed. Using littermates for controls helps to reduce genetical heterogeneity. In living organisms it cannot be ruled out that compensating mechanisms make it more difficult to elucidate the role of a lacking peptide or protein, which might also result in reduced effect strength. On top of these problems many KO mouse models, like PS1/2 double KO or APP/APLPL2 double KO mice, are lethal in embryonic or early postnatal status (Wang et al., 2005a). For AICD transcriptional regulation of proteins involved in tissue differentiation are postulated (see Table 2). Especially the lethal phenotype of mice lacking APP and APLP2 in early stages aggravates the analysis of AICD function on gene transcription of these proteins.

In addition to the above mentioned KO systems, a truncated APP construct expressed in absence of the whole APP family is used in some studies (Huysseune et al., 2009; Grimm et al., 2011a,b). The truncated APP lacks the CTF and therefore AICD. By utilizing this system it is in principal possible to distinguish if an effect is caused by AICD or other APP cleavage products. However, the missing CTF also influences Aβ generation making this differentiation less distinct. To avoid or circumvent this problem it might be helpful to test if the observed phenotype could be partially rescued by adding AICD peptide, combining the advantages and disadvantages of both systems. Additionally the analysis of the KO or knock down of AICD adaptor proteins like Fe65 might help to understand the underlying mechanism, especially in combination with other experimental approaches.

Finally, promoter analysis using, e.g., luciferase assays should be mentioned. Utilizing this approach it is possible to investigate if AICD is indeed able to affect the specific promotor regions of potential target genes. It has to be taken into consideration that these systems mainly use episomal vectors, making transport, or transport dependent processes to the nucleus unnecessary. This experimental approach is not suitable to distinguish between the transcriptional impact of C83 (α-CTF) and C99 (β-CTF) derived AICD.

As no optimal experimental approach having no disadvantages is available, it has to be accepted that only the combination of different experimental methods and models is sufficient to unambiguously evaluate the role of AICD in gene regulation.

#### Summary

The combination of several genetic and/or pharmalogical approaches is necessary to elucidate the AICD-mediated regulation of potential AICD target genes. For genes found to be regulated by AICD in cell culture systems the *in vivo* relevance should be tested in animal models, and to rule out in general an effect of AICD several tissues have to be analyzed as the gene regulatory role of AICD seems to be tissue specific.

### Gene regulatory functions of APP independent of AICD

Besides AICD, it has been reported that other APP cleavage products and even full length APP have an impact on gene regulation.

The secreted APP ectodomain possibly regulates downstream target genes via binding to one or several yet unknown receptor(s) activating intracellular pathways leading to altered gene expression. sAPPα was shown to increase the expression of several neuroprotective genes, among them transthyretin (TTR) and insulin-like growth factor-2 (IGF-2) in mouse organotypic hippocampal cultures and protects them from Aβ-induced tau phosphorylation and neuronal death (Stein et al., 2004). Recently, a study by Ryan et al. (2013) confirmed such a function of sAPPα in rat hippocampal organotypic slice cultures, where sAPPα rapidly elicited a multi-level transcriptional response including the regulation of several transcription factors, microRNAs and the modulation of the chromatin environment. The kinase CDK5 and the chaperone ORP150 have been also reported to be regulated by sAPPα. Treatment of neurons with sAPPα peptide leads to reduced expression and activity of CDK5, which is involved in the phosphorylation of tau and induces Aβ generation (Cruz et al., 2006; Piedrahita et al., 2010). In contrast, the expression of the neuroprotective chaperone ORP150 (Kitao et al., 2001; Tamatani et al., 2001) is induced by sAPPα treatment. Importantly, these effects could not be observed in *Sorl1*-deficient neurons arguing for a role of SORLA as an essential sAPPα receptor (Hartl et al., 2013). In APP/APLP2-deficient mice the expression of TTR, involved in amyloid suppression (Schwarzman et al., 1994; Choi et al., 2007), and Klotho, related to various aging processes (Kuro-o et al., 1997; Kurosu et al., 2005) is downregulated. In contrast, an upregulation of TTR and Klotho mRNA levels was observed in sAPPβ-knockin mice indicating a role of the APP β-cleaved ectodomain in the regulation of these genes (Li et al., 2010).

Independent of sAPPα, sAPPβ, and AICD generation APP has been reported to regulate gene expression as well. Transcriptional downregulation of acetylcholinesterase (AchE) by full length APP was found in two neuronal cell lines (Hicks et al., 2013). Additionally, APP holoprotein was reported to regulate cholesterol metabolism at a transcriptional level. Pierrot et al. (2013) showed the expression of APP in rat cortical neurons to decrease both the mRNA levels of 3-hydroxy-3-methylglutaryl-coenzym-A-reductase (HMGCR) and cholesterol 24-hydroxylase leading to a reduction in cholesterol turnover and to inhibition of neuronal activity. APP was further reported to downregulate the expression of the transcription factor EGR-1 at both mRNA and protein levels *in vivo* and in cultured neurons and in a γ-secretase-independent manner (Hendrickx et al., 2013). Additionally, intracellular Aβ peptide is discussed to act as transcription factor binding to Aβ peptide interacting domain (AβID) sequences within the promoter regions of some genes. Ohyagi et al. (2005) showed intracellular Aβ to directly activate the p53 promoter resulting in p53-dependent apoptosis. The transcription factors ASCL1 and OLIG2 are also regulated in cell culture by Aβ (Uchida, JBC, 2007). Moreover, interactions between Aβ and sequences within the APP and BACE1 promoters have been reported by the use of Chip assays on human neuroblastoma cells (Bailey et al., 2011) and by electrophoretic mobility shift assays (EMSA) indicating that Aβ peptide may regulate genes involved in its own production (Maloney and Lahiri, 2011). However, further studies concerning the role of APP and its cleavage products in gene regulation are necessary.

#### Summary

Impact in gene regulation is not only reported for AICD, but also for full length APP and its cleavage products sAPPα, sAPPβ, and Aβ.

## Concluding Remarks

Summing it up, several lines of evidence underline the importance of NEP in Aβ clearance. A reduced NEP activity has been associated with AD and increased Aβ levels. In return upregulation of NEP might be protective or beneficial for AD. However, the underlying mechanisms of NEP regulation are controversially discussed. One of the most favored models suggests that the amyloidogenic APP cleavage product, AICD, which has high similarities to Notch, is involved in the transcriptional regulation of NEP. Nevertheless additional studies will be necessary to further clarify the role of AICD in NEP regulation and to elucidate whether indeed NEP upregulation is suitable to prevent or treat AD. As AICD has been linked to the induction of genes both involved in neuroprotection and neurotoxicity, an upregulation of AICD can not be assumed as positive in general. On the one hand, AICD is discussed to upregulate the expression and/or activity of APP, BACE1, p53, and GSK3β leading to enhanced Aβ generation, apoptosis and tau phosphorylation, all of these processes linked to neurotoxicity. On the other hand, AICD induces NEP expression leading to increased Aβ degradation resulting in lowered Aβ levels. Regarding the therapeutic potential of an AICD upregulation further studies are necessary to answer the question if the positive or negative consequences of an AICD upregulation predominate. Moreover, the site effects of an enhanced NEP level also need further investigation keeping in mind the numerous substrates of NEP including several neuropeptides.

**Figure 5:**
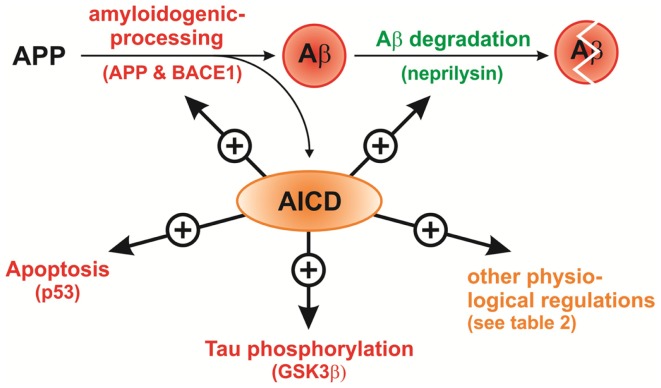
**Regulation of genes involved in neuroprotection (green), neurotoxicity (red) or other genes (orange) by AICD**. On the one hand AICD enhances (+) the expression and/or activity of APP, the β-secretase BACE1, p53, and GSK3β leading to enhanced Aβ generation, apoptosis, and tau phosphorylation. On the other hand AICD also induces NEP gene expression resulting in increased Aβ degradation and reduced Aβ levels. A regulatory cycle can be postulated, in which AICD regulates its own production via induction of APP and BACE1 gene expression also generating Aβ peptide, whose degradation is stimulated at the same time via upregulation of NEP expression.

A regulatory cycle can be postulated, in which AICD regulates its own production via induction of APP and BACE1 gene expression with the Aβ peptide as secondary product, whose degradation is stimulated at the same time via upregulation of NEP gene expression (summarized in Figure 5). In a pathological situation like AD this cycle seems to be disturbed resulting in enhanced Aβ production along with reduced NEP levels leading to the severe accumulation of Aβ in brain tissue.

## Author Contributions

Marcus O. W. Grimm, Janine Mett, Christoph P. Stahlmann, Viola J. Haupenthal, Valerie C. Zimmer, and Tobias Hartmann wrote the manuscript.

## Conflict of Interest Statement

The authors declare that the research was conducted in the absence of any commercial or financial relationships that could be construed as a potential conflict of interest.
